# Auto-Tandem Catalytic Reductive Hydroformylation in a CO_2_-Switchable Solvent System

**DOI:** 10.1021/acssuschemeng.2c00419

**Published:** 2022-03-08

**Authors:** Sebastian Püschel, Jan Sadowski, Thorsten Rösler, Kira Ruth Ehmann, Andreas J. Vorholt, Walter Leitner

**Affiliations:** † Max Planck Institute for Chemical Energy Conversion, 45470 Mülheim an der Ruhr, Germany; ‡ Institute for Technical and Macromolecular Chemistry, RWTH Aachen University, 52074 Aachen, Germany

**Keywords:** reductive hydroformylation, switchable solvent system, tandem catalysis, synthetic fuels

## Abstract

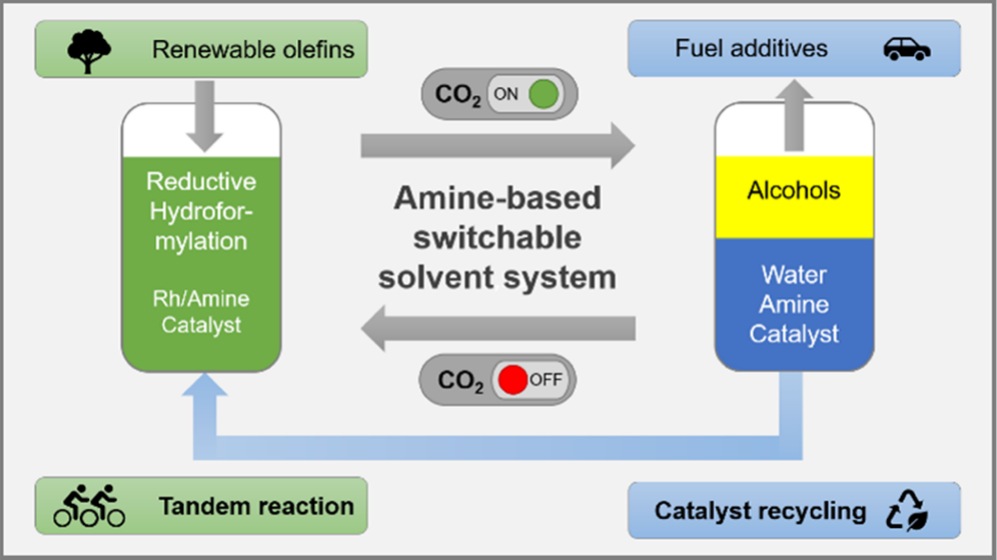

Upgradation of olefin-enriched Fischer–Tropsch cuts by the synthesis of alcohols leads to drop-in-capable biosynthetic fuels with low carbon emissions. As an alternative to the conventional two-step production of long-chain alcohols, tandem catalytic systems improve the energy and resource efficiency. Herein, we present an auto-tandem catalytic system for the production of alcohols from olefin–paraffin mixtures. By utilization of a tertiary alkanolamine as the ligand as well as the switchable component in the solvent system, a lean reaction system capable of catalyst recycling was developed. The system was characterized with regard to the switchable solvent separation approach and reaction parameters, resulting in alcohol yields of up to 99.5% and turnover frequencies of up to 764 h^–1^. By recycling the catalyst in 10 consecutive reactions, a total turnover number of 2810 was achieved.

## Abstract 2

Double utilization of alkanolamines: As nonphosphine ligand for tandem catalysis and for switching the phase behavior for product separation

## Introduction

As an immediate measure to reduce carbon dioxide emissions of road transport, biosynthetic fuels enable the ongoing use of existing infrastructure and vehicles.^1−3^ One pathway toward renewable fuels is olefin-selective Fischer–Tropsch (FT) synthesis with bio-syngas.^4^ The FT-derived olefins allow for subsequent upgrading of such fuels and enable tailor-made fuel properties. Potential additives to biosynthetic fuels are alcohols, which are able to reduce emissions and improve combustion characteristics while aiding to achieve drop-in capability.^5−7^

Alcohols in the C_6_–C_11_ range (for diesel requirements) are industrially produced in multistep processes, predominantly in hydroformylation–hydrogenation reactions with isolated aldehyde intermediates.^8,9^ Aldehydes represent platform chemicals in industry and lead to various products, including carboxylic acids, amines, and alcohols. For fuel production, direct production of alcohols without intermediate steps could increase the resource efficiency.^10,11^

Direct conversion of olefins to alcohols is known as “reductive hydroformylation”, an example of tandem catalysis and the combination of a hydroformylation and a hydrogenation step. Several tandem catalytic systems for this conversion have been investigated: separate catalysts (orthogonal tandem catalysis) for either reaction step^12,13^ and so-called “assisted tandem catalysis” in which both steps are conducted with the same catalyst, although under different reaction conditions.^14^ However, auto-tandem catalysis—using the same catalyst for both reaction steps under the same conditions—represents the most efficient variant.^11^ Auto-tandem reductive hydroformylation was first described for cobalt-based catalysts, although they achieve poor selectivity and require harsh conditions.^15,16^ Recent investigations usually focus on rhodium catalysts due to their superior activity in hydroformylation, which are capable of auto-tandem reductive hydroformylation, e.g., in combination with tertiary alkylamines.^17−24^ Necessary steric and electronic properties of amines in reductive hydroformylation were previously published by our group.^21,25^

However, most existing tandem catalytic systems for this reaction inherit the most fundamental challenge in homogeneous catalysis: difficult recovery of the expensive catalyst(s). A rather unconventional approach to catalyst recycling is the so-called “switchable solvent system”.^26^ In this system, the addition of a trigger (commonly CO_2_) to a switchable component changes its ionic strength and therefore the phase behavior of the mixture.^27^

**Figure 1:**
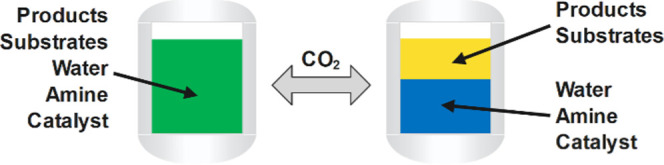
General concept of the CO_2_-switchable phase behavior.

Figure 1 shows the process concept of the switchable phase behavior with the addition of CO_2_ to an amine. However, the reaction is carried out under monophasic conditions (Figure 1, left) and product separation is achieved by the addition of CO_2_, which causes the mixture to separate into two liquid phases (Figure 1, right).

This effect eliminates the necessity of additional solvents for extraction and their respective energy-intensive recovery and further increases the efficiency of a tandem catalytic system.^27−29^ In this manuscript, we present a water/alkanolamine-based CO_2_-switchable solvent system for reductive hydroformylation to yield alcohols from olefins in one step.

The alkanolamine serves two functions in this system: it is part of the catalyst system (ligand) and also serves as a switchable component (solvent) to influence the phase behavior of the reaction mixture. Given the polar nature of the catalyst (ionic rhodium complexes and a polar amine), the catalyst accumulates in the lower (polar) phase, and the nonpolar reaction products accumulate in the upper (organic) phase. Hence, this technique enables the recovery of the catalyst.

## Results and Discussion

**Scheme 1:**
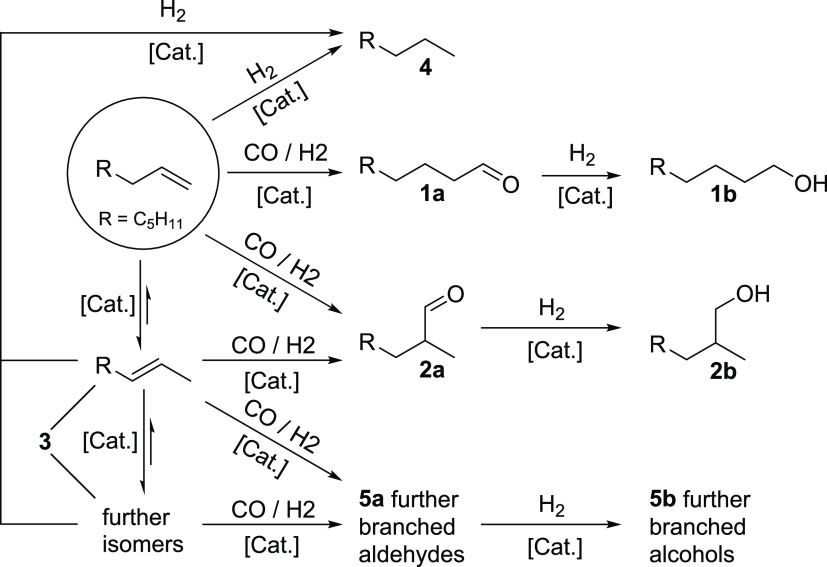
Reductive Hydroformylation Reaction Network

Scheme 1 shows the reductive hydroformylation reaction network. As a representation of FT cuts, an olefin–paraffin mixture was used as the model substrate, consisting of 1-octene and *n*-heptane (1:1 mass ratio). The olefins react to either linear (**1a**) or branched (**2a**) aldehydes in the first reaction step. Possible side reactions are the formation of internal, isomerized olefins (**3**) as well as hydrogenation of the olefins to the corresponding paraffin (**4**); however, in all experiments, only traces of paraffins were found, an exceptional selectivity compared to cobalt-based catalyst systems.

Hydroformylation of isomerized olefins (**3**) leads to further branched aldehydes (**5a**). In the second reaction step, all intermediate aldehydes (**1a** + **2a** + **5a**) are hydrogenated to their corresponding alcohols (**1b** + **2b** + **5b**), which represent the desired reaction products. To assess the first reaction step, the combined hydroformylation yield is used (*Y*_hyfo_, **1a** + **1b** + **2a** + **2b** + **5a** + **5b**). The total alcohol yield (*Y*_alcohols_, **1b** + **2b** + **5b**) is used to evaluate the second reaction step.

The necessary properties of amines in reductive hydroformylation reactions have been determined in previous contributions by our group. Therein, catalytically active species were characterized with operando spectroscopy. Furthermore, the basicity of the amine was determined to be an important parameter; a p*K*_a_ value of 10 ± 1.5 is necessary for the production of alcohols.^21,25^ Also, the steric hindrance of the employed amines needs to be low, and bulky groups on the amines as well as diamines reduced the hydrogenation activity of the system. This disables the control of the regioselectivity of the reactions with bulky ligand systems. However, this is of minor importance for this reaction system; the desired reaction products are primary alcohols as fuel additives, which are not required to be linear.

To avoid reductive amination reactions,^18,30^ only tertiary amines are applicable in reductive hydroformylation. Furthermore, the tertiary alkylamines used in previous studies^21^ show poor performance in CO_2_ capture and therefore are not suitable for designing a switchable solvent system.

Instead, alkanolamines are necessary to achieve reasonable “switching power”.^28^ Given the aforementioned constraints for amines in reductive hydroformylation, the selection of amines applicable in the reaction as well as separation is rather limited. *N*,*N*-Dimethylethanolamine (DMAE) and *N*,*N*-diethylethanolamine (DEAE) were identified as suitable polar amine candidates for this reaction.

### Investigation of Phase Separation

DMAE led to the undesired formation of solids when pressurized with CO_2_. On the contrary, when DEAE was used, no formation of solids occurred and hence was used as the switchable component.

To develop a reaction/phase separation system, first the separation step was characterized. In a phase behavior simulation with Aspen Plus (included in the Supporting Information, SI), a water–amine ratio (WAR, *m*_water_/*m*_DEAE_) of ≤0.5 was found to be suitable for monophasic behavior during the reaction. These results were validated experimentally by generating potential reaction mixtures with the pure substances present in the reaction (water, DEAE, *n*-heptane, 1-octene, and *n*-nonanol). Table 1 summarizes the phase behavior of these simulated reaction mixtures. Since amphiphilic alcohols are produced from nonpolar substrates, the alcohol yield has a large influence on the phase behavior of the reaction mixture.

**Table 1:** Number of Phases at a Given Surrogate Mixture Composition^a^

*Y*_alcohols_	WAR 0	WAR 0.1	WAR 0.2	WAR 0.3	WAR 0.4	WAR 0.5
0.00	1	1	2	2	2	2
0.25	1	1	2	2	2	2
0.50	1	1	1	1	1	1
0.75	1	1	1	1	1	1

a Water–amine ratio (WAR, *m*_water_/*m*_DEAE_), *Y*_alcohols_ simulated by replacing 1-octene with *n*-nonanal.

Generally, low water content of the mixture leads to monophasic behavior for any alcohol yield, *i.e.*, already at the outset of the reaction. With an increased water–amine ratio, these polar components form a separate phase immiscible with the substrate; the initially present two phases merge when a sufficient alcohol concentration is reached.

As a water-less system (WAR = 0) would potentially eliminate any solvent in the reaction system, it would be the most efficient option. Unfortunately, water is necessary for the amine to switch to the ionic form; the reaction network of the protonation of tertiary amines involves carbonate and bicarbonate ions.^31^

**Figure 2:**
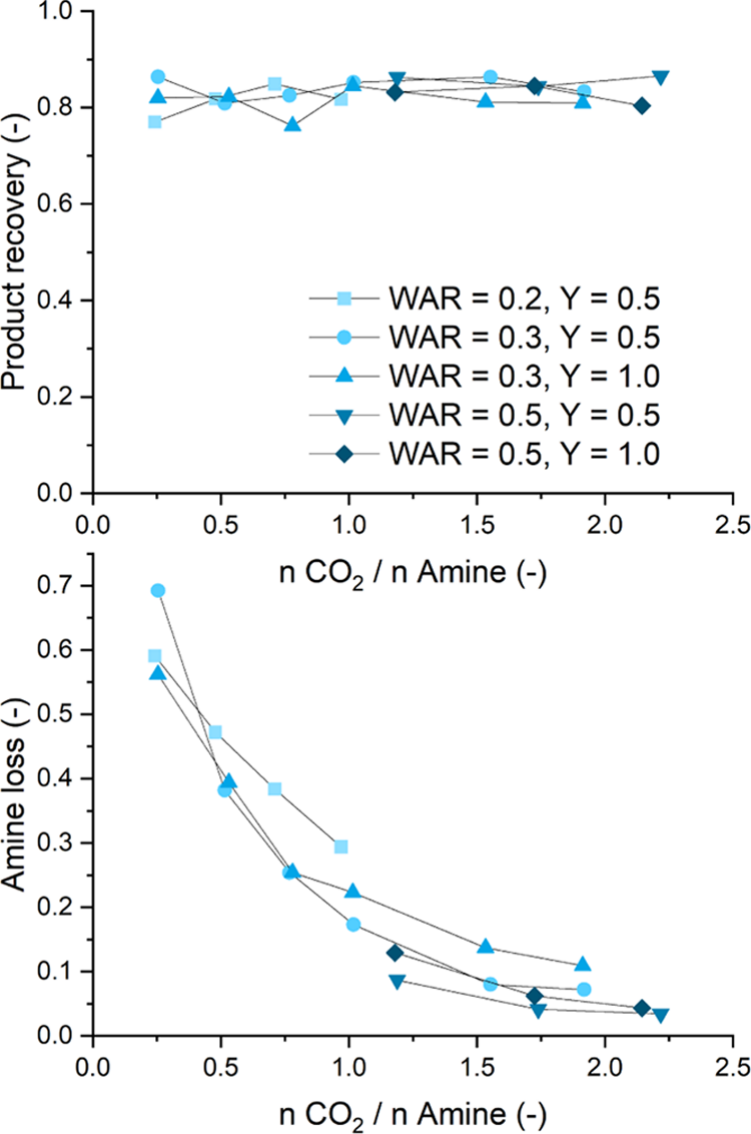
Product recovery and amine leaching with varying WAR and CO_2_ loading. Separation conditions: *T*_sep_ = 25 °C, *t*_sep_ = 5 min, product recovery = *n*_alcohol, second phase_/*n*_alcohol, total_, water–amine ratio (WAR) = *m*_Water_/*m*_DEAE_, and *Y* = simulated alcohol yield in the surrogate mixture.

Accordingly, no reaction between CO_2_ and the amine was observed when no water was added to the mixture. Hence, water–amine ratios between 0.1 and 0.5 were considered for further investigations of the phase separation performance (Figure 2).

To investigate the phase behavior and compositions of the formed phases after pressurization with CO_2_, surrogate mixtures, the previously described mixtures, were pressurized with varying molar ratios between CO_2_ and DEAE. Figure 2 shows that while the product recovery remains similar for any variation of the parameters, the amount of amine lost into the product phase decreases with higher CO_2_ loading. This can be attributed to increased ionic strength of the catalyst phase since a higher conversion of DEAE into the protonated form is achieved at high CO_2_ loading. The loss of amine also decreases slightly with increased water content of the polar phase.

### Batch Optimization of Reaction Conditions

**Figure 3:**
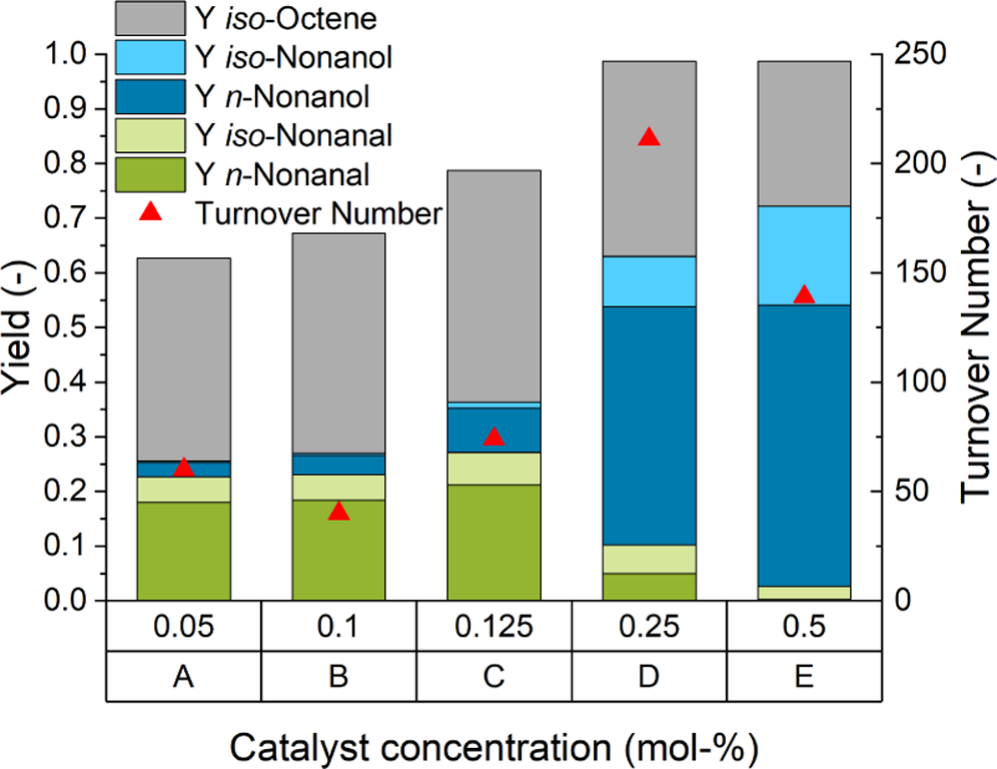
Variation of catalyst concentration. Reaction conditions: *n*_1-octene_ = 38.4 mmol (6 mL), *V*_*n*-heptane_ = 6 mL, *T* = 100 °C, *t*_R_ = 1.5 h, *p* = 30 bar, CO/H_2_ = 1:2, φ_org_ = 0.4, *V*_liq_ = 30 mL, water–amine ratio = 0.3, *r* = 2000 min^–1^, and catalyst = [Rh(acac)(CO)_2_].

The initial reaction conditions for the presented reaction system were adapted from a previously published system developed by our group in which the reaction mixture consisted of acetonitrile as the solvent and diethylmethylamine was used as the amine ligand.^21^ These components were replaced by water and *N*,*N*-diethylethanolamine, respectively. With the solvent system modified to allow for CO_2_-switchable behavior, similar activity and alcohol yield were obtained in an initial experiment (Figure 3E, *Y*_alcohols_ = 70%, compared to 82% in the reference system).

Given the high catalyst loading necessary for this reaction, one main goal of the investigation was to reduce the required amount of rhodium. At a catalyst concentration of 0.25 mol %, comparable yields were achieved (Figure 3D), leading to a higher turnover number (TON_alc_, mol alcohols produced per mol of catalyst) of 211 compared to 139 with 0.5 mol %. Further reduction of the catalyst loading led to decreased yield and lower productivity (TON_alc_) of the catalyst. In particular, the hydrogenation activity of the catalyst system was significantly reduced at lower catalyst loadings (Figure 3A–C). A catalyst concentration of 0.25 mol % has therefore been used for further investigation of this reaction system.

To allow for the desired CO_2_-switchable behavior of the system, the solvent as well as the amine were replaced with compounds with increased polarity. While this is necessary to separate the nonpolar reaction products from a now polar catalyst phase, the solubility of the nonpolar gaseous substrates as well is expected to be low in this reaction system.^32^

**Figure 4:**
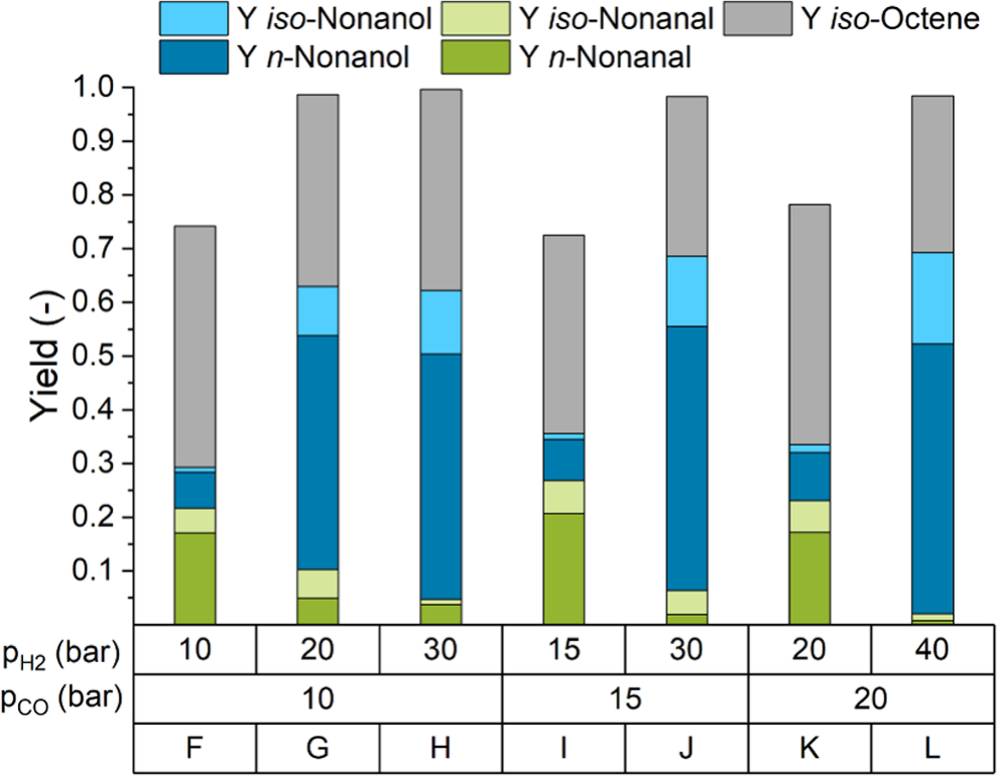
Variation of Syngas pressure and composition. Reaction conditions: *c*_cat_ = 0.25 mol %, *n*_1-octene_ = 38.4 mmol (6 mL), *n*_Rh_ = 0.096 mmol, *V*_*n*-heptane_ = 6 mL, *T* = 100 °C, *t*_R_ = 1.5 h, φ_org_ = 0.4, *V*_liq_ = 30 mL, water–amine ratio = 0.3, *r* = 2000 min^–1^, and catalyst = [Rh(acac)(CO)_2_].

Hence, the influence of the syngas pressure and composition was investigated. A stoichiometric composition (CO/H_2_ = 1:2 for the tandem reaction) led to higher selectivity toward alcohols compared to a ratio of 1:1 (Figure 4F,I,K).

Hydrogen excess (CO/H_2_ = 1:3, Figure 4H) not only leads to similar results with a slightly higher hydrogenation activity but also an increased total pressure. Hence, a ratio of CO/H_2_ = 1:2 and a total pressure of 60 bar were chosen for the following experiments.

**Figure 5:**
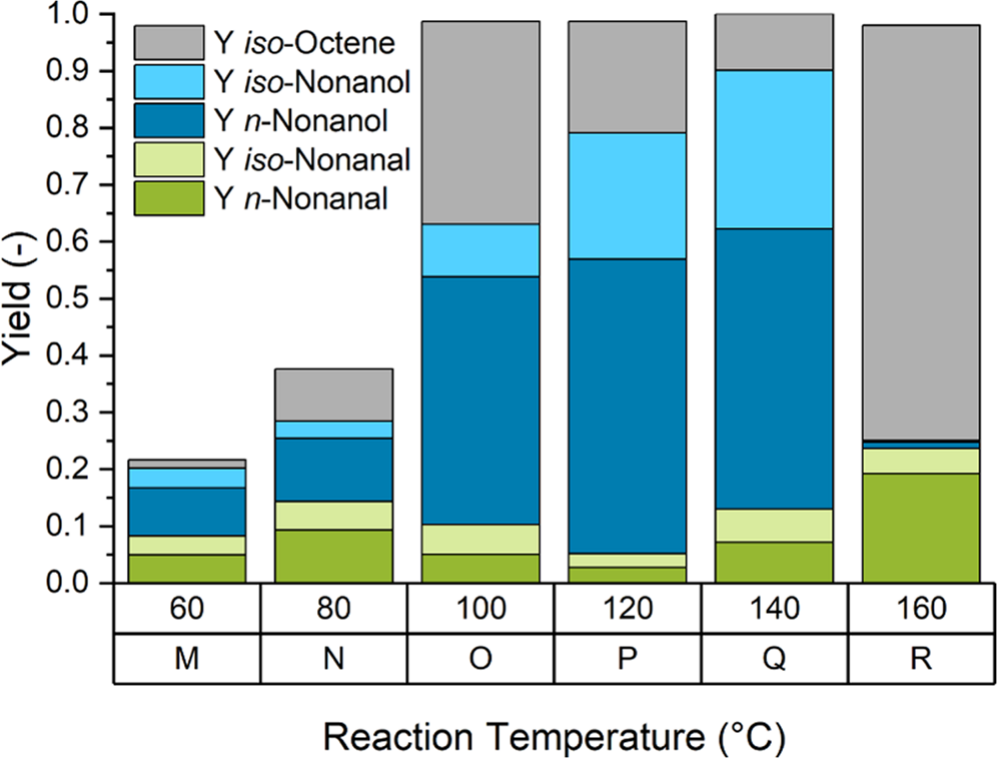
Variation of the reaction temperature. Reaction conditions: *c*_cat_ = 0.25 mol %, *n*_1-octene_ = 38.4 mmol (6 mL), *n*_Rh_ = 0.096 mmol, *V*_*n*-heptane_ = 6 mL, *p* = 60 bar, CO/H_2_ = 1:2, *t*_R_ = 1.5 h, φ_org_ = 0.4, *V*_liq_ = 30 mL, water–amine ratio = 0.3, *r* = 2000 min^–1^, and catalyst = [Rh(acac)(CO)_2_].

Furthermore, the effect of the reaction temperature was investigated. Lowering the reaction temperature to 60 or 80 °C significantly reduced the activity of the catalyst (Figure 5M,N). Higher reaction temperatures of 120 and 140 °C led to an increased alcohol yield (Figure 5P,Q). Primarily, this can be attributed to the hydroformylation of isooctene (**3**) to branched aldehydes (**5a**) and the subsequent production of branched alcohols (**5b**), which is apparently accelerated at higher temperatures. At 160 °C, isomerized olefins were observed to be the predominant reaction products. At the same time, the catalyst activity for hydrogenation is almost completely abolished. This phenomenon is already visible when increasing the temperature from 120 to 140 °C: the hydrogenation activity decreases and a higher share of aldehyde intermediate remains in the reaction product.

Most likely, the catalytic species active for hydrogenation are no longer stable at these high temperatures.

Furthermore, the water–amine ratio, important for the phase behavior of the reaction (Table 1), was evaluated using time profile experiments to compare the individual reaction steps. As shown, when characterizing the phase behavior, increased water/amine ratios of ≥0.2 lead to a biphasic start of the reaction and therefore initially cause mass transfer limitations between the two liquid phases. Hence, a reduced initial reaction rate could be expected.

**Figure 6:**
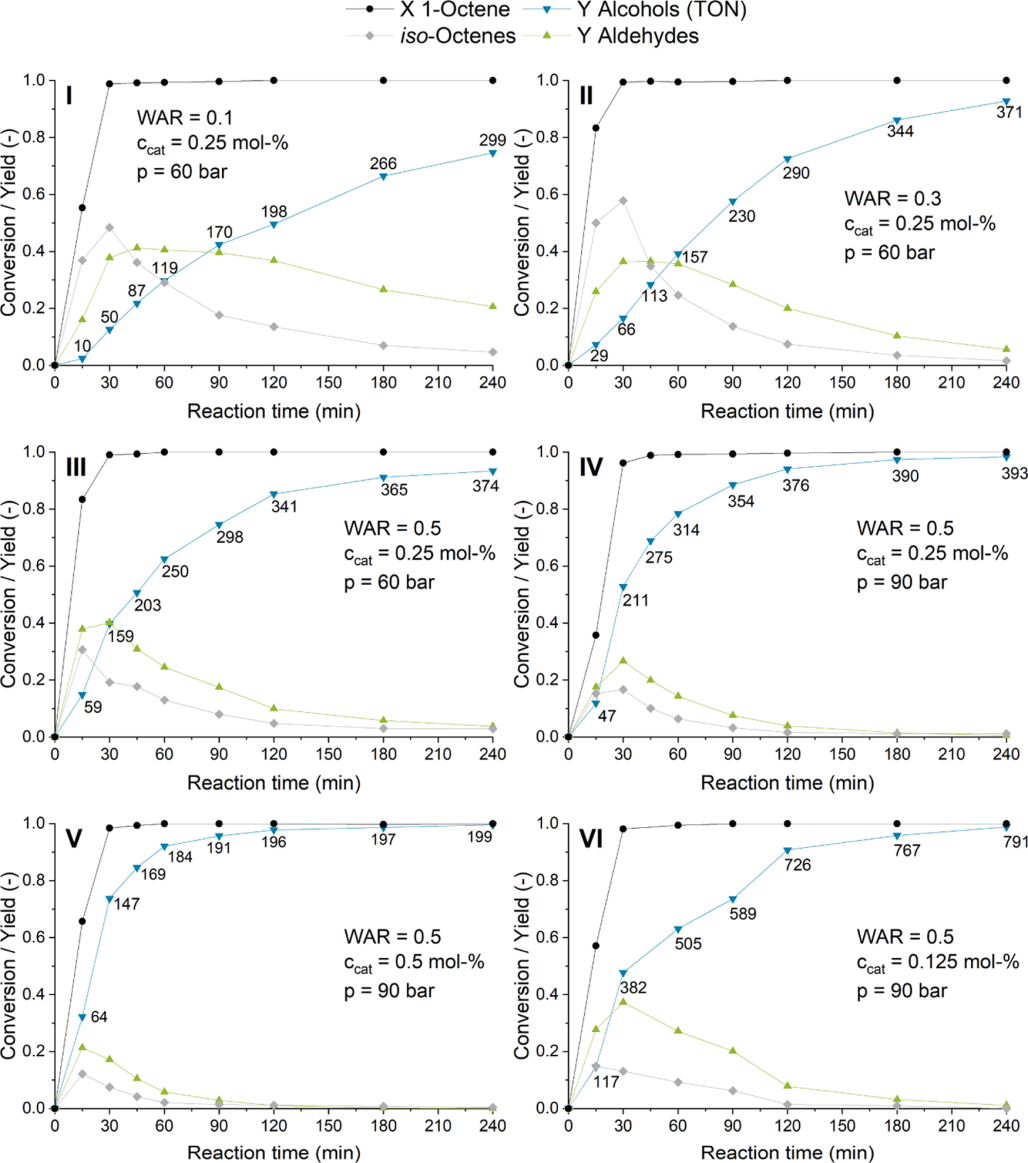
Time profile experiments with varying water/amine ratios, syngas pressure, and catalyst concentration. Conditions for all graphs: reaction conditions: *n*_1-octene_ = 166.4 mmol (26 mL), *n*_Rh_ = 0.416 mmol, *V*_*n*-heptane_ = 26 mL, *T* = 140 °C, CO/H_2_ = 1:2, φ_org_ = 0.4, *V*_liq_ = 130 mL, *r* = 2000 min^–1^, and catalyst = [Rh(acac)(CO)_2_].

On the contrary, the reaction to alcohols was found to be faster at a water–amine ratio of 0.5 compared to lower ratios (Figure 6I–III). After 90 min, an alcohol yield of 75% was observed. This value increased to 93% after 4 h. Compared to a water–amine ratio of 0.3, less olefin isomers are formed during the first 30 min of the reaction. Potentially, this is due to a lower accessibility of the catalyst for the substrate due to the described mass transfer limitations. Consequently, all following experiments were carried out with a WAR of 0.5.

To suppress the formation of internal olefins even further, a pressure increase to 90 bar was investigated. At this pressure, the yield of olefin isomers reached a maximum of only 16% at 30 min (Figure 6IV) compared to 30% at 60 bar (Figure 6III). Furthermore, aldehyde hydrogenation is also accelerated at 90 bar, likely because of an increased concentration of the gaseous substrate in the catalyst phase. The combination of 90 bar and a WAR of 0.5 lead to an overall alcohol yield of 98.4% after 4 h. The initial reaction was also increased, and a turnover frequency (TOF_alc_) of 422 h^–1^ was detected in the first 30 min.

The effect of different catalyst concentrations was investigated in time profiles as well (Figure 6V,VI). While the overall reaction rate is the highest at 0.5 mol %, the catalytic productivity (TON_alc_) is increased at lower catalyst loadings. All investigated catalyst concentrations led to almost quantitative yield after 4 h; hence, the lowest catalyst concentration leads to maximum catalytic productivity. At 0.125 mol %, the highest TON_alc_ (791) as well as the highest initial TOF_alc_ (764 h^–1^ in the first 30 min) were achieved, which represents the highest catalytic activity for rhodium/amine-catalyzed auto-tandem reductive hydroformylation reported so far.^21,33^

### Catalyst Recycling

**Figure 7:**
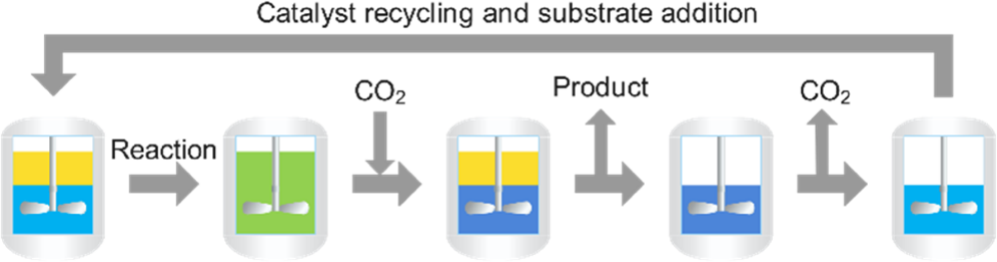
Switchable solvent system catalyst recycling concept.

After investigating the separation and reaction parameters, the gained knowledge was applied in catalyst recycling experiments. The procedure of catalyst recycling in this switchable solvent system is displayed in Figure 7.

The system starts as a biphasic mixture (at high water–amine ratios of ≥0.2), which turns into a single phase due to the formation of alcohols (Table 1 and Figure 2). The reaction mixture is then pressurized with CO_2_ to switch to a liquid–liquid biphasic system. In this generated biphasic mixture, the products accumulate in the upper phase and can be separated by simple decantation. To reuse the catalyst phase in another reaction run, CO_2_ must be removed from the amine to restore the initial reaction conditions for the next cycle.

**Figure 8:**
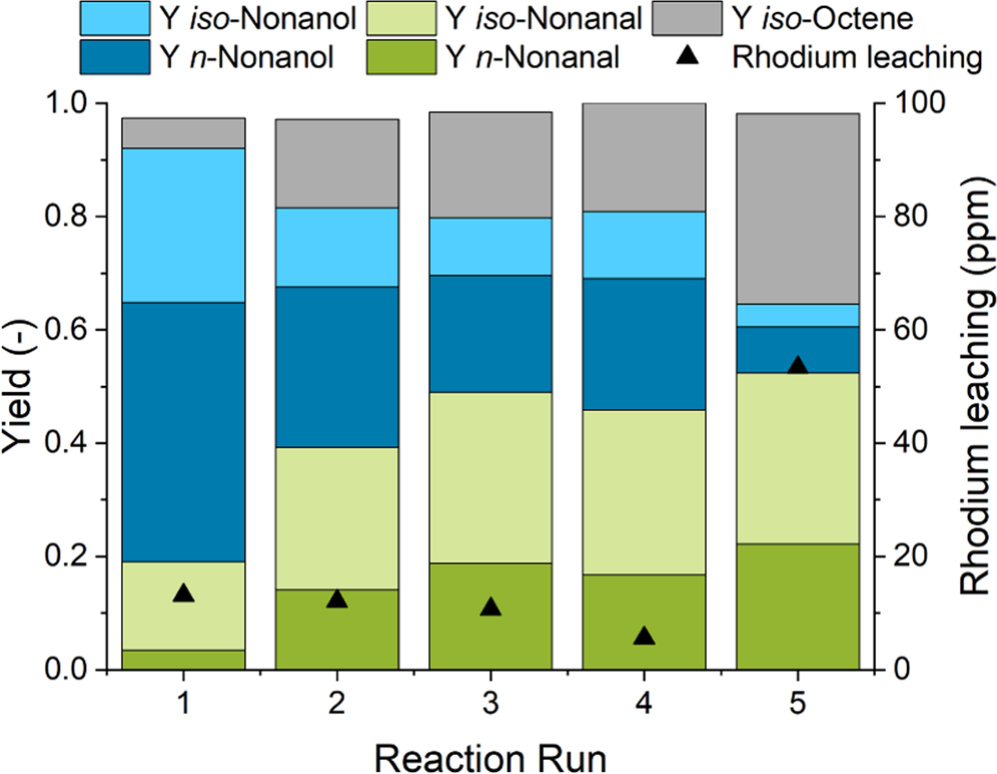
Proof-of-concept recycling experiment. Reaction conditions: *c*_cat_ = 0.25 mol %, *n*_1-octene_ = 166.4 mmol (26 mL), *n*_Rh_ = 0.416 mmol, *V*_*n*-heptane_ = 26 mL, *T* = 140 °C, *p* = 90 bar, CO/H_2_ = 1:2, *t*_R_ = 1.5 h, φ_org_ = 0.4, *V*_liq_ = 130 mL, water–amine ratio = 0.5, *r* = 2000 min^–1^, and catalyst = [Rh(acac)(CO)_2_]; separation conditions: *T*_sep_ = 25 °C, *t*_sep_ = 5 min, *n*_CO_2__/*n*_DEAE_ = 2.0, *T*_rev_ = 70 °C, and *t*_rev_ = 20 min.

In a first proof-of-concept experiment (Figure 8) for the described recycling approach, the slow release of CO_2_ from DEAE at standard conditions and the air stability of tertiary amines were utilized to conduct the phase separation outside of the reactor in a separatory funnel. As this experiment was carried out using 0.25 mol % of the catalyst, the achieved total turnover number of 773 corresponds to an approximate twofold increase of catalytic productivity; the maximum TON_alc_ achievable in a single batch reaction would be 400. This proves the feasibility of the catalyst recycling concept. However, the alcohol yield drops after each run, suggesting the presence of catalyst leaching or deactivation.

Hence, inductively coupled plasma mass spectrometry (ICP-MS) measurements were conducted to quantify the rhodium concentration in the organic product phase. In the first four runs, a rhodium leaching of 5–15 ppm was detected. However, in the fifth run, catalyst leaching increased to 53 ppm (5.2% of 8.1% in total); at the same time, a strong decrease of catalyst activity was observed. This leads to the assumption that nonpolar catalyst species were formed, which are especially less active for aldehyde hydrogenation. Potentially, this was caused either by repeated air contact during the separation or the absence of a CO_2_ atmosphere during the actual phase separation. Hence, for the following experiments, a more sophisticated reaction setup allowing the separation under CO_2_ pressure was used. The setup is further described in the Supporting Information.

To increase the ionic strength of the catalyst phase and to induce phase separation, DEAE is converted into a protonated compound. Likely, this protonated species is incapable of stabilizing the ionic rhodium species, reported as the active catalyst during previous investigations.^21,34,35^ Virtually removing the amine from the reaction mixture to switch the phase behavior could explain the deactivation of the catalyst in this experiment.

**Figure 9:**
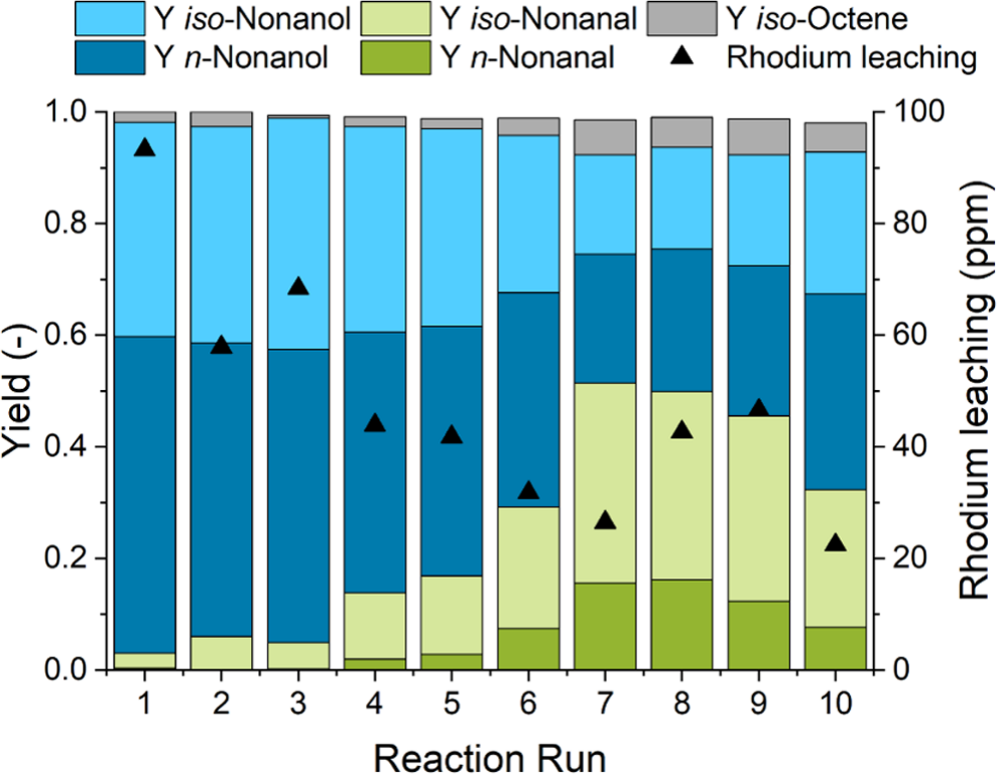
Recycling experiment with reduced CO_2_ loading in phase separation. Reaction conditions: *c*_cat_ = 0.25 mol %, *n*_1-octene_ = 166.4 mmol (26 mL), *n*_Rh_ = 0.416 mmol, *V*_*n*-heptane_ = 26 mL, *T* = 140 °C, *p* = 90 bar, CO/H_2_ = 1:2, *t*_R_ = 3 h, φ_org_ = 0.4, *V*_liq_ = 50 mL, water–amine ratio = 0.5, *n* = 2000 min^–1^, and catalyst = [Rh(acac)(CO)_2_]; separation conditions: *T*_sep_ = 25 °C, *t*_sep_ = 5 min, *n*_CO_2__/*n*_DEAE_ = 0.9, *T*_rev_ = 70 °C, and *t*_rev_ = 20 min.

To circumvent this phenomenon, a recycling experiment with a deficiency of CO_2_ compared to the amine (*n*_CO_2__/*n*_DEAE_ = 0.9) was conducted. This leads to the presence of nonprotonated DEAE during phase separation, which should stabilize the catalyst. Figure 9 indicates that this approach improves the stability of the catalyst. Compared to the experiment in Figure 8, this recycling experiment resulted in stable catalytic activity over at least three reaction runs. From reaction run 4 onward, low aldehyde hydrogenation activity is observable, which causes an increased share of the intermediate in the reaction product. In total, a TTON_alc_ of 2810 for the auto-tandem production of alcohols was achieved in this experiment.

However, as already observed in the phase separation experiments (Figure 2), increased amine loss into the organic phase follows from the reduced ratio between CO_2_ and amine. Hence, a makeup of amine is required and was performed in this experiment. This loss of amine furthermore is an explanation for the increased catalyst leaching observed in this experiment. Interestingly, more catalyst is lost during this experiment (37% in total), but the activity of the catalyst was maintained for a longer period of time. This suggests the presence of an additional deactivation mechanism linked to the CO_2_-induced phase switching, leading to a tradeoff between the activity of the catalyst and the efficiency of the recycling concept. This effect should be further characterized using operando spectroscopy in future investigations.

## Conclusions

In this work, a switchable solvent approach for reductive hydroformylation was investigated in batch and recycling experiments. The use of *N*,*N*-diethylaminoethanol as the switchable component as well as the catalyst ligand and water as the only additional solvent led to a lean system for auto-tandem reductive hydroformylation. By characterizing the main influence parameters, the switchable solvent approach for catalyst recycling was proven to be feasible. The system showed a strong dependency on the water–amine ratio present in the reaction as well as the CO_2_–amine ratio applied during phase separation. After optimization in batch experiments, the reaction system achieved alcohol yields of up to 99%. Furthermore, a maximum turnover number of 791 in a single batch reaction was detected and an initial turnover frequency of 764 h^–1^ (during the first 30 min) was achieved. To the best of our knowledge, this is the most active rhodium-based auto-tandem system for reductive hydroformylation reported so far. After investigation of the reaction and separation technique regarding catalytic stability, recycling the catalyst nine times was possible. The utilization of the catalyst was improved, achieving a TTON_alc_ of 2810 for tandem catalytic alcohol production. Further improvement of the catalytic stability is accessible by investigations using operando spectroscopy. Advanced process control of the separation conditions in an improved reaction setup could potentially reduce the loss of amine and therefore reduce catalyst loss and deactivation.

## Supporting Information Available

Detailed experimental protocol and information on the used equipment, additional information on the phase behavior simulation, and IR spectra of CO_2_ loaded and regenerated reaction mixture (PDF)
