# Supplementary material for: Auto-Tandem Catalytic Reductive Hydroformylation in a CO2-Switchable Solvent System
Source: ACS Sustain Chem Eng. 2022 Mar 8;10(11):3749–56. doi: 10.1021/acssuschemeng.2c00419 (PMC8942186; doi:10.1021/acssuschemeng.2c00419)
Supplement: Supplementary file 1 — sc2c00419_si_001.pdf [file sc2c00419_si_001.pdf]

Supplementary Information

Auto-tandem catalytic reductive hydroformylation in a CO2-switchable solvent system

Sebastian Püschel[a], Jan Sadowski[a], Thorsten Rösler[a], Kira Ruth Ehmann[a], Andreas J. Vorholt[*,a], Walter Leitner[a,b]

[a] Max Planck Institute for Chemical Energy Conversion, 45470 Mülheim an der Ruhr, Germany

[b] Institute for Technical and Macromolecular Chemistry, RWTH Aachen University, 52074 Aachen, Germany

* Email: andreas-j.vorholt@cec.mpg.de

Number of pages: 6

Figures

Figure S1. Technical drawing of the used Parr Instruments custom autoclave........................S2
Figure S2. Example for a calibration curve. In this case for DEAE and the use of a flame ionization detector with 1-heptanol as internal standard...S4
Figure S3. Water-Amine-Ratio phase behavior simulation using Aspen Plus. Modeled with UNIFAC, T = 25°C, Product recovery = $n_{alcohol, second\ phase}$ / $n_{alcohol, total}$ S5
Figure S4. IR spectra recorded before (black) and during (green and red) pressurization and after removal of CO_2 (blue) S6

Tables

Table S1. Gas chromatography calibration factors for FID and TCD.......................................S4

Experimental

Chemicals

1-octene (99+%), n-heptanol (98%) and [Rh(acac)(CO)$_2$] (98.5%) were acquired from Acros Organics, n-heptane (99+%) and 2-propanol (99+%) were purchased from Carl Roth GmbH & Co. KG. *N*,*N*-diethylaminoethanol (DEAE, >99.5%), was obtained from Sigma Aldrich. A Merck Milli-Q® IQ purification system prepared Ultrapure water (conductivity <0,055 µS cm-1, TOC <3 ppb,). Hydrogen (99,999%), Carbon monoxide (99,997%) and Carbon dioxide (99,995%) were supplied by Westfalen AG.

Experimental setup

The experiments were conducted using a Parr Instruments 4560 high-pressure stainless-steel autoclave with either the standard 300 mL reactor or a custom-made 250 mL windowed reaction vessel for observation of phase behaviour. Both vessels are heated by an electrical heating jacket and stirred by a mechanically driven 4 pitched blade agitator. Precise dosing of all gaseous components was achieved by using Bronkhorst High-Tech B.V CORI-FLOW™ mass flow controllers. A technical drawing of the reactor is displayed in Figure S1. For the recycling experiment displayed in Figure 9 of the main document, three draft tubes at different heights inside the reactor allowed for the removal of the organic product phase even with different positions of the phase interface.

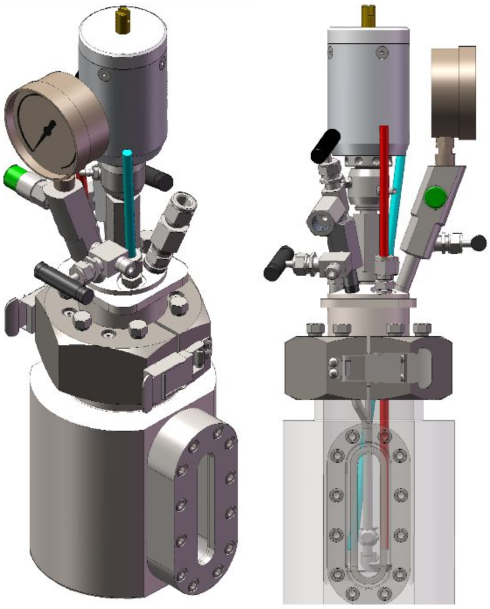

Figure S1. Technical drawing of the used Parr Instruments custom autoclave.

Experimental procedure

[Rh(acac)(CO)2] was stored and weighed in an oxygen- and water-free glovebox. All liquid components were purged with argon before use. First, the catalyst was dissolved in DEAE and Milli-Q water under standard Schlenk conditions. Argon counterflow was applied when filling catalyst and substrate solutions into the reactor. Afterwards the reactor was pressurized with carbon monoxide and hydrogen and heated to the desired temperature. During heat-up, the reactor was stirred at 150 min^{-1} to improve heat transfer.

The reaction was started by increasing the stirring rate to 2000 min^{-1} after reaction temperature was reached. After the reaction, unconverted syngas was released and the reactor was purged with argon in order to remove any remaining toxic or flammable gas.

In phase separation experiments (Figure 2 in the main document), prepared mixtures generated from the individual components were filled into the reactor. Subsequently, the desired amount of CO_2 was filled into the reactor using a Bronkhorst High-Tech B.V CORI-FLOW™ mass flow controller. For the absorption of CO_2 into the mixture, a stirrer rate of 1000 min^{-1} was applied to facilitate mass transfer into the liquid phase. The reactor was further equipped with a Bronkhorst High-Tech B.V EL-Press pressure sensor, which allowed for observation and recording of the pressure inside the reactor. This device was used to observe the process of the absorption of CO_2 into the mixture, which occurred rather quickly. A constant pressure was observed after approximately 2 minutes of stirring. In order to achieve reproduceable results, this time was set to t_{sep} = 5 minutes for any experiment. Any phase separation was carried out at T_{sep} = 25°C.

After these 5 minutes, the CO_2 remaining in the gas phase was removed from the reactor through an exhaust gas line. Subsequently, the reactor was opened and the now biphasic mixture was transferred into a separatory funnel for precise phase separation. The two obtained phases were then weighed and their respective composition was determined using gas chromatography. (see Analytics)

The catalyst recycling experiment displayed in Figure 8 of the main document was conducted using a similar procedure, however the phase separation was carried out by just tilting the reactor and thereby removing the organic product phase. This was possible with sufficient precision due to the high viscosity of the CO_2-loaded polar phase, which adhered to the reactor wall and therefore remained in the reaction vessel. This method was chosen to minimize the loss of catalyst phase.

As stated above, the reactor was equipped with a total of three draft tubes for the experiment in Figure 9 of the main document, which allowed for the removal of a large share of the organic product phase even though the position of the phase interface changed over the course of the experiment (due to amine loss and a minor accumulation of the produced alcohols in the lower phase). This setup allowed for the separation under CO_2 pressure, which represents an improvement of the reproducibility of the process.

For both types of recycling experiments, the CO_2 content of the catalyst phase needed to be removed before starting the next reaction run in order to achieve the desired phase behavior. As the release of CO_2 from aqueous amine solutions is rather slow at ambient conditions (this behavior was utilized in the experimental procedure described above), the reactor was heated to T_{rev} = 70°C to facilitate the reverse reaction (release of CO_2). As the reactor was equipped with a window, the formation of gas bubbles could be observed. Furthermore, the equilibrium was shifted further away from the ionic species by purging the solution with an argon stream. Depending on the CO_2 loading, the formation of bubbles stopped after 5 to 10 minutes of stirring (150 min^{-1}) at 70°C. Hence, the timeframe for the reverse reaction was chosen to be t_{rev} = 20 min.

In the experiment where the separation was carried out under CO_2 pressure, the organic phase was removed using a draft tube. Afterwards, the CO_2 release procedure was started. Subsequently, fresh substrate solution (1-octene and n-heptane) was fed using a HPLC pump (Flusys WADose Lite HP) to avoid any contamination of the catalyst phase with air. Afterwards, the reactor was heated to the desired reaction temperature and the next reaction run was started.

Analytics

A Shimadzu Nexis GC 2030 gas chromatograph with a flame-ionization-detector (FID) and an additional thermal conductivity detector (TCD) was used to determine all yields. Both lines were equipped with a Restek Corp. RTX 5 polysiloxane column with 30 m length, 1 µm film thickness and 0.25 mm internal diameter. Hydrogen was used as carrier gas. Injection of samples was carried out via a Shimadzu AOC 20iPlus injection system with a Shimadzu AOC 20sPlus autosampler.

For analysis, samples (175 mg) were diluted with 2-propanol (800 mg). n-heptanol (25 mg) was used as internal standard. The individual factors obtained in the calibration are given in Table S1. An example for such a calibration curve is given in Figure S2.

For the calibration curves, a linear equation of the form y = m*x+b was applied, were b was defined as zero. Hence, only "m" is given in Table S1.

Table S1. Gas chromatography calibration factors for FID and TCD.

Component	m (FID)	R^2 (FID)	m (TCD)	R^2 (TCD)
1-Octene	0.86827	0.99995	1.02893	0.99996
i-Octene (= 1-Octene)	0.86827	0.99995	1.02893	0.99996
n-Octane	0.91565	0.9990	1.02893	0.99890
n-Nonanal	1.05226	0.99996	1.0896	0.99985
i-Nonanal (= n-Nonanal)	1.05226	0.99996	1.0896	0.99985
n-Nonanol	0.9579	0.99980	1.0446	0.99969
i-Nonanol (= n-Nonanol)	0.9579	0.99980	1.0446	0.99969
n-Heptane	0.91565	0.99959	1.05524	0.99964
DEAE	1.2411	0.9994	1.1318	0.9992
Water	-	-	0.7088	0.99382

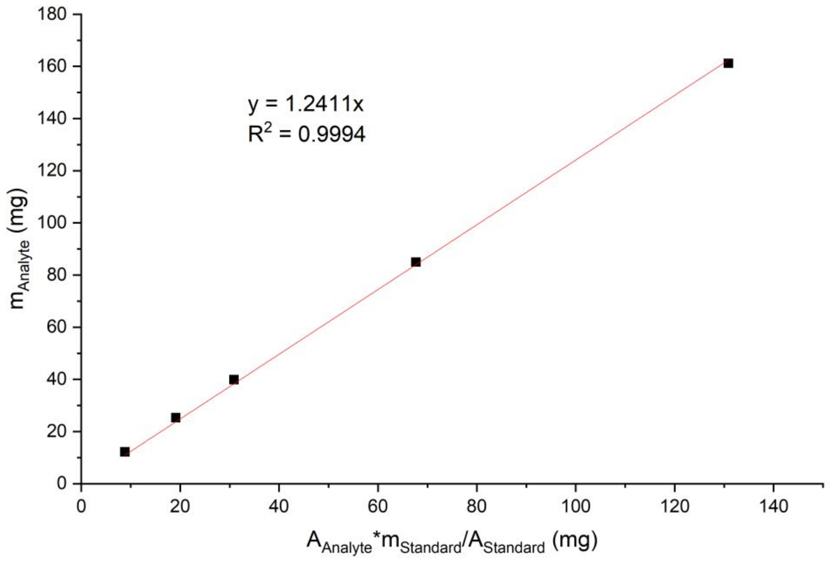

Figure S2. Example for a calibration curve. In this case for DEAE and the use of a flame ionization detector with 1-heptanol as internal standard.

Given the switching of the phase behavior, different samples were analyzed in different types of the discussed experiments. As stated above, in the phase separation experiments (Figure 2 in the main document), both phases obtained in the experiment were analyzed using GC. During the optimization of the reaction parameters in batch and time profile experiments, a single sample of the monophasic reaction mixture was analyzed for the determination the yield of the individual components, no phase separation was conducted.

In recycling experiments (Figures 8 and 9 in the main document), a sample of the monophasic reaction mixture was taken before the phase separation was conducted. This sample served for the detection of the yields. After the phase separation, the organic phase was removed from the reactor and subsequently also analyzed using GC in order to determine the product recovery and the loss of amine *via* mass balance calculations.

Inductively Coupled Plasma - Mass Spectrometry (ICP-MS) was performed with a Shimadzu ICPMS-2030. ICP-MS samples were digested with nitric acid in a CEM Corp. Mars 6 microwave reactor prior to measurement.

Phase behavior simulation

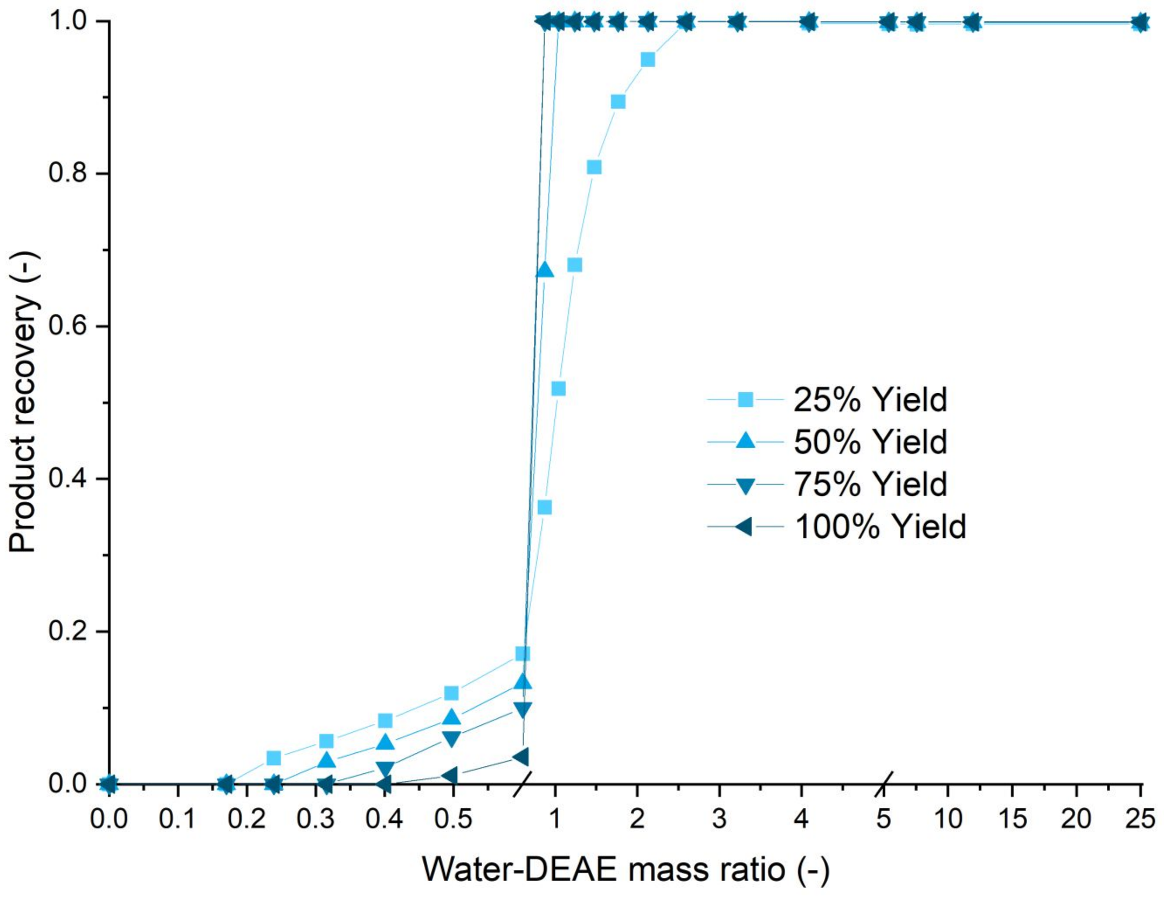

Figure S3. Water-Amine-Ratio phase behavior simulation using Aspen Plus.
Modeled with UNIFAC, T = 25°C, Product recovery = $n_{alcohol, second\ phase}$ / $n_{alcohol, total}$

The phase behavior simulation with Aspen Plus shows a switch of phase behavior approximately at equal water and amine mass fractions. When using a catalyst phase with higher water fractions (high Water-Amine-ratio), a second, organic phase is present throughout the reaction. If the product recovery is zero, no second phase is formed. Hence, using an excess of amine in the reaction leads to monophasic behavior, especially at high alcohol yields, eliminating liquid-liquid mass transfer limitations.

CO_2 removal and confirming ATR-IR experiments

For the measurements displayed below, an ArcSpectro FTIR-FC-4TE IR device obtained from ArcOptix was used, which was connected to an ATR-IR probe with a diamond prism, manufactured by IFS Infrared Fiber Sensors Aachen.

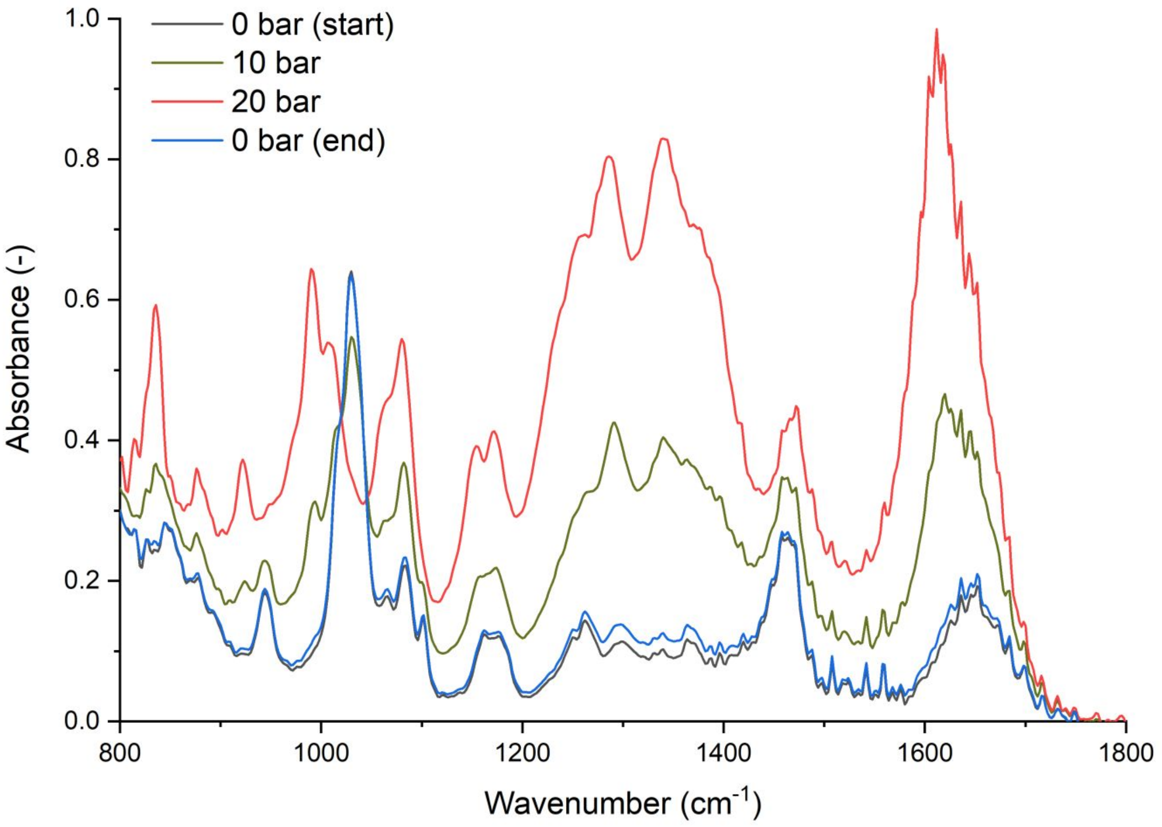

Figure S4. IR spectra recorded before (black) and during (green and red) pressurization and after removal of CO_2 (blue)

As Figure S4 indicates, the effect of the pressurization of the mixture is clearly visible when using IR spectroscopy. Furthermore, the effective removal of the CO_2 applied can be confirmed by this method, as the black spectrum (before the pressurization) and blue spectrum (after the removal of CO_2 from the mixture using the procedure described above) are well matched.

Furthermore, the successful restoration of the initial conditions could also be observed through the window of the reaction vessel. The phase separation occurs almost immediately after CO_2 is fed to the reactor. When removing the CO_2 from the mixture, after 5 to 10 minutes, no formation of gas bubbles can be observed any more and the mixture becomes monophasic once again.
